# 

**Published:** 1879-11-20

**Authors:** 


### TABLE OF CONTENTS.

ORIGINAL AND SELECTED ARTICLES.

Aborted Croupus Pneumonia, by A. G. Hobbs, M.D., Indiana, 401. Enlargement of the Right Testis in Child Two Years of Age, by Lindsay Johnson, M.D., Demonstrator of Anatomy in Southern Medical College, 403. Aconite in Pneumonia, by A. C. F. Rabagliati, M.A., M.P., 404. Hypodermic Use of Morphia, 406. Phytolacca Decandra as a Remedial Agent in Mastitis, by Wm. F. Holt, M.D., Macon, 408. Alcohol in Fever, 409. Letter from Vienna—Cæsarean Section, 411. The Therapeutic Value Croton Chloral, 412. New Mixture, 413. The Stigmata of Maize and the Arenaria Rubra in Diseases of the Bladder, 414. Management of Retained Secundines in Abortion, 415. Good Effects of Ammoniacal Sulphate of Copper in Neuralgia of the Fifth Nerve (Tic Douloureux), 416. Dextro-Quinine in Pertussis, by J. L. Coombs, M.D., Grass Valley, Cal., 417.

ABSTRACTS AND GLEANINGS.

Post Partum Hemorrhage, 419. Therapeutic Effects of the Subcutaneous Injection of Iron, Condurango, etc., 420. Ovariotomy, etc., 421. After Delivery, 423. Treatment of Cardiac Dyspnœa, 424. Uterine and Vaginal Applications, 425. Tattooing of Nævi, 426. Skin Irritation by the Administration of Drugs; New Treatment of Ozæna, 427. An Effective Cathartic; Hot Water in Chancroids; Case of Foreign Body in the Stomach, 428. Gingivitis of Puerperal Women; Nervous Vomiting—Electricity; The Tattooing of Cutaneous Nævi, 429. Phosphate of Lime; Syphilitic Neuralgia; Treatment of Albuminuria; Glycerine in Phthisis, 430.

SCIENTIFIC ITEMS.

Moral Dietetics; The Mound Builders; Alexis St. Martin, 431. Origin of Plants; Anticipation of the Microphone; Gnoscopine; Petrolina and Petrolina Oil, 432.

PRACTICAL NOTES AND FORMULÆ.

Fever in a Child; Anteflexion of the Uterus, 433. Post Nasal Catarrh; Ophthalmia Neonatorum; Liquid Glue, 434. Treatment of Barber's Itch; Popp's Anatherine Dentifrice; Lip Salve; Glycerole of Thymol, 435. Remedial Management of Acute Testitis; Ulcers and Abrasions; Russian Spirit; Ptyalism, 436.

EDITORIAL AND MISCELLANEOUS.

Notice; Editorial Notices; Surgeon-General J. K. Barnes, 437. Duty on Quinine; Medical Societies, 438. Recent Additions to the Medical Talent of Atlanta; Donations to College Library; Book Notices, 439. Receipted; Special Notices, 440.

